# Potent Antimicrobial and Antibiofilm Activities of Feleucin-K3 Analogs Modified by α-(4-Pentenyl)-Ala against Multidrug-Resistant Bacteria

**DOI:** 10.3390/biom11050761

**Published:** 2021-05-19

**Authors:** Xiaomin Guo, Tiantian Yan, Jing Rao, Xin Yue, Xiong Pei, Jiahui Deng, Wangsheng Sun, Wenle Yang, Bangzhi Zhang, Junqiu Xie

**Affiliations:** Key Laboratory of Preclinical Study for New Drugs of Gansu Province, School of Basic Medical Sciences & Research Unit of Peptide Science, Chinese Academy of Medical Sciences, 2019RU066, Lanzhou University, Lanzhou 730000, China; guoxm17@lzu.edu.cn (X.G.); yantt19@lzu.edu.cn (T.Y.); raoj18@lzu.edu.cn (J.R.); yuex20@lzu.edu.cn (X.Y.); peix18@lzu.edu.cn (X.P.); dengjh18@lzu.edu.cn (J.D.); sunws@lzu.edu.cn (W.S.); yangwl@lzu.edu.cn (W.Y.); zhangbz@lzu.edu.cn (B.Z.)

**Keywords:** antimicrobial peptide analogs, unnatural hydrophobic amino acid, α-(4-pentenyl)-Ala, antimicrobial activity, antibiofilm activity

## Abstract

The dramatic increase in antimicrobial resistance (AMR) highlights an urgent need to develop new antimicrobial therapies. Thus, antimicrobial peptides (AMPs) have emerged as promising novel antibiotic alternatives. Feleucin-K3 is an amphiphilic α-helical nonapeptide that has powerful antimicrobial activity. In our previous study, it was found that the fourth residue of Feleucin-K3 is important for antimicrobial activity. After α-(4-pentenyl)-Ala was introduced into this position, both the antimicrobial activity and stability were greatly improved. Herein, to improve the limitations of Feleucin-K3, this unnatural amino acid was further introduced into different positions of Feleucin-K3. Among these synthetic Feleucin-K3 analogs, the N-terminal-substituted analog Feleucin-K65 (K65) and C-terminal-substituted analog Feleucin-K70 (K70) had preferable antimicrobial activity. In particular, their antimicrobial activities against multidrug-resistant bacteria were more potent than that of antibiotics. The stabilities of these peptides in salt and serum environments were improved compared with those of Feleucin-K3. In addition, these analogs had low hemolytic activity and AMR. More importantly, they effectively inhibited biofilm formation and exhibited considerable efficacy compared with traditional antibiotics against biofilm infection caused by methicillin-resistant *Staphylococcus aureus* (MRSA). In antimicrobial mechanism studies, K65 and K70 mainly permeated the outer membrane and depolarized the cytoplasmic membrane, resulting in cellular component leakage and cell death. In summary, analogs K65 and K70 are potential antimicrobial alternatives to solve the antibiotic crisis.

## 1. Introduction

The development of antibiotic drugs has greatly reduced the incidence and mortality of bacterial infections [1]. However, the overreliance on antibiotics has aggravated the antimicrobial resistance (AMR), thereby seriously threatening global public health [2]. Antimicrobial peptides (AMPs) have broad-spectrum antimicrobial activity due to their rapid bactericidal ability and mechanism of action, which is mainly through nonspecific membrane destruction, attracting widespread attention as promising new antibiotic candidates [3]. In addition, the formation of biofilms has always been considered one of the main reasons by which bacteria develop resistance, making it more difficult for conventional antibiotics to treat bacterial infections [4]. There are reports that AMPs can inhibit and eradicate biofilms to treat biofilm-related infections [5]. However, some disadvantages of AMPs, such as high toxicity and poor proteolytic stability, limit their clinical applications [6].

The introduction of unnatural amino acids has always been one of the most common strategies to improve the limitations of the AMPs mentioned above. Unnatural amino acids are not recognized by proteases, which increase the proteolysis resistance compared with natural amino acids [7]. Unnatural amino acids may increase the antimicrobial activity by balancing amphiphilicity with hydrophobicity [8] and may stabilize the structural conformation with intramolecular interactions to improve the salt tolerance [7]. For example, Wang et al. [9] described an increase in AMP salt resistance and activity after replacing tryptophan residues with β-naphthylalanine, which contains a hydrophobic naphthyl group on its side chain.

Feleucin-K3 (FLKLLKKLL-NH_2_), a cationic nonapeptide composed of lysine (Lys or K), phenylalanine (Phe or F) and leucine (Leu or L) residues, is a core sequence analog of an AMP secreted by the skin of *Bombina orientalis* [10]. It adopts a typical amphiphilic α-helical structure and displays powerful antimicrobial activity against a range of bacteria, especially *Pseudomonas aeruginosa* (*P. aeruginosa*), which comprises a large proportion of the bacteria isolated in the clinic [11]. Hence, Feleucin-K3 is worthy of further study. Structure–activity relationship studies have demonstrated that the key site is the fourth residue, and the antimicrobial activity shows significant enhancement when it is replaced with alanine [11]. In addition, after α-(4-pentenyl)-Ala, an unnatural hydrophobic amino acid, was introduced as the fourth residue, both the antimicrobial activity and stability improved greatly [12].

In this study, to maximize the antimicrobial activity and stability while minimizing the toxicity, the unnatural amino acid α-(4-pentenyl)-Ala was further introduced into different positions of Feleucin-K3. Thus, Feleucin-K3 analogs with α-(4-pentenyl)-Ala substitutions were designed and synthesized. Their antibacterial activity, antibiofilm activity, stability, toxicity and ability to induce AMR were comprehensively investigated. Among these analogs, K65 and K70 were screened, and their possible antimicrobial mechanisms were explored. Finally, the antibiofilm activities of the ideal short peptides K65 and K70 in vivo were determined through a biofilm infection model.

## 2. Materials and Methods

### 2.1. Peptide Synthesis

The Feleucin-K3 analogs in our study were synthesized by a stepwise solid-phase peptide synthesis (SPPS). The molecular weights of the analogs were confirmed by electrospray ionization mass spectrometry (ESI-MS; maXis 4G, Bruker Daltonics, Bremen, Germany). The crude peptides were purified by a preparative reversed-phase high-pressure liquid chromatography (RP-HPLC; Waters 600, Milford, MA, USA). Analytical RP-HPLC was used to determine the purity of the analogs, which should be more than 95%.

### 2.2. Strains and Animals

All sensitive bacteria and multidrug-resistant bacteria were obtained from the Key Laboratory of New Drug Preclinical Research of Gansu Province, Lanzhou University, Lanzhou, China.

Female BALB/c mice were provided by the Medical Experimental Animal Center of Lanzhou University (SYXK GAN 2018-0002). The animal experiments strictly complied with the Guide for the Care and Use of Laboratory Animals of the National Institutes of Health and were approved by the Ethics Committee of Lanzhou University.

### 2.3. Circular Dichroism (CD) Spectroscopy

The α-helical structures of the peptides were investigated using a J-810 spectrometer (Jasco, Tokyo, Japan). Peptides (100 μg/mL) were dissolved in 50% trifluoroethanol (TFE) to mimic a hydrophobic membrane environment and 0.01-M PBS to mimic an aqueous environment [13]. The spectral wavelengths ranged from 190 to 260 nm. The other parameters were as follows: 50-nm/min scanning rate, 1-nm bandwidth and a 1-s response.

### 2.4. Antimicrobial Activity and Salt Stability In Vitro

The minimum inhibitory concentration (MIC) of the Feleucin-K3 analogs was tested by the broth microdilution method [11]. Sensitive bacteria and drug-resistant bacteria were diluted to 1 × 10^6^ CFU/mL, according to the McFarland standards. Bacterial suspension and peptide solutions (8–128 μg/mL) were incubated at 37 °C for 18 h. The minimum concentration without obvious bacterial growth was the MIC of the peptides. The stability of the analogs in a physiological salt environment was also investigated. In brief, salt powder (450-mM NaCl) was dissolved in deionized water, and the following investigational steps were the same as those described before. The above steps were repeated several times, until the same value was obtained three times.

### 2.5. Hemolytic Activity

Mouse red blood cells (RBCs) were collected using the eyeball removal method to investigate the hemolytic activity of the Feleucin-K3 analogs [14]. Then, these analogs were incubated with the same volume of 8% RBC suspension for 1 h [11]. After centrifugation for 10 min, 100 μL of supernatant was withdrawn, and the optical density at 490 nm was detected. The hemolysis rate of the cells treated with 0.1% Triton X-100 (Solarbio, Beijing, China) was used as a positive control, and PBS was used as a negative control.

### 2.6. Serum Stability

The Feleucin-K3 analogs (10 mM) were mixed with human serum (Biotop, Beijing, China) in a 1:4 ratio and incubated at 37 °C. At different times, cold acetonitrile was used to terminate the reaction. After centrifugation for 10 min, the remaining peptides were monitored by RP-HPLC. The elution gradient was 5%/95% acetonitrile/water.

### 2.7. In Vitro Antibiofilm Activity

The antibiofilm activity of the peptides was determined according to the previously described methods [15]. Strains (final concentration of 5 × 10^5^ CFU/mL) and peptides (4–128 μg/mL) were incubated for 24 h at 37 °C. Biofilm cells were cleaned with 0.01-M PBS and fixed with methanol. Crystal violet (0.1%, HopeBiol, Qingdao, China) was used to stain the biofilm cells for 10 min. After washing with water, 95% ethanol was finally added, and the optical density at 595 nm was measured with a microplate reader (Flex Station 3; Laguna Hills, CA, USA).

### 2.8. Confocal Laser Scanning Fluorescence Microscopy (CLSM)

The antibiofilm activity of the Feleucin-K3 analogs was determined using a LIVE/DEAD Biofilm Viability Kit and concanavalin A conjugates (Invitrogen, Waltham, MA, USA). In brief, MRSA ATCC 33591 (5 × 10^5^ CFU/mL) and an equal volume of peptides (final concentration 4 μg/mL or 8 μg/mL) were incubated in culture dishes. Propidium iodide (PI) at the concentration of 6 μg/mL and the same concentration of SYTO9 were premixed and added to the dish [16]. After incubation for 30 min, CLSM (Zeiss LSM 710 Meta; Karl, Germany) was used to visually observe the results. The three-dimensional structure images were obtained by Z-stack mode. In addition, the exopolysaccharide matrix secreted by the biofilm cells was stained with concanavalin A conjugates (40 μg/mL) and observed according to the above method.

### 2.9. Scanning Electron Microscopy (SEM)

MRSA ATCC 33591 (5 × 10^5^ CFU/mL) and peptides (low concentration of 4 μg/mL or high concentration of 8 μg/mL) were incubated for 24 h. Then, cell climbing sheets with biofilms were gradually processed according to the following steps: washing with PBS several times, fixing with 2.5% glutaraldehyde overnight at 4 °C, dehydration in ethanol for 10 min, freeze-drying for 6 h, coating in gold for 30 s and, finally, observing with an Apreo S (Thermo Fisher Scientific, Waltham, MA, USA) [17]. In addition, the morphological changes in the MRSA treated with peptides were also observed using the Apreo S. MRSA ATCC 33591 (1 × 10^9^ CFU/mL) was incubated with analogs (2 × MIC or 4 × MIC) for 30 min or 120 min. The cells were processed and observed as previously described.

### 2.10. Time-Killing Assay

The rapid bactericidal ability of the Feleucin-K3 analogs was evaluated by time-killing kinetics [18]. K65 and K70 were diluted to different concentrations ranging from 1 × to 4 × MIC. Bacteria (5 × 10^5^ CFU/mL) were mixed with K65 and K70. After incubation in a 1:1 ratio, the number of bacteria at different times was determined by plating on Mueller–Hinton agar plates.

### 2.11. Resistance Development Assay

Serial passage and MIC determination were used to explore whether the bacteria developed drug resistance. On the first day, the MICs of the peptides were determined as described above. The next day, the bacterial suspension of sub-MIC concentration was cultivated to the mid-log phase. The MICs of the peptides were detected again, and this process was repeated for 20 days. The MICs of the antibiotics, including amoxicillin (AML), ceftazidime (CAZ) and imipenem, were also determined as controls.

### 2.12. Lipopolysaccharide (LPS)/Lipoteichoic Acid (LTA) Competitive Inhibition Assay

An equal volume of LPS/LTA (2–1024 μg/mL) was mixed with K65 or K70 (2 × MIC) [19]. After incubation for 1 h, the bacterial suspension (5 × 10^5^ CFU/mL) was added, followed by coincubation for another 2 h. Finally, the samples were properly diluted, and the number of colonies was recorded by plating on Mueller–Hinton agar plates.

### 2.13. Outer Membrane Permeabilization

The bacterial outer membrane permeabilization induced by the Feleucin-K3 analogs was determined using the fluorescent dye *N*-phenyl-1-naphthylamine (NPN; J&K Scientific, Beijing, China) [20]. In brief, mid-log-phase bacteria were centrifuged and resuspended to an OD_600_ = 0.5 in HEPES buffer. Different concentrations of K65 and K70 were added first, followed by the addition of NPN (40 μM) and the prepared bacterial suspension. The fluorescence changes within 20 min were monitored using a microplate reader.

### 2.14. Cytoplasmic Membrane Depolarization

Bacterial cytoplasmic membrane depolarization was investigated using 3,3′-dipropylthiadicarbocyanine iodide (DiSC_3_(5); Sigma Aldrich, St Louis, MO, USA) [20]. In short, a bacterial suspension (OD_600_ = 0.1) was resuspended in HEPES buffer containing 5-mM HEPES, 20-mM glucose and 100-mM KCl. DiSC_3_(5) (4 μM) and the bacteria were incubated at 37 °C for 1 h. Then, this mixture and peptides were added to a 96-well plate. The fluorescence was monitored in real time using a microplate reader.

### 2.15. PI Uptake Assay

A PI uptake assay was used to explore the membrane integrity after treatment with the peptides [20]. Mid-log-phase *S. aureus* and *Acinetobacter baumannii* (*A. baumannii*) were diluted to 1 × 10^8^ CFU/mL. Then, the bacteria were treated with K65 and K70 (4 × MIC) for 30 min. PI dye (1 mg/mL) was added, followed by incubation for another 15 min. The samples were observed by CLSM.

### 2.16. DNA-Binding Affinity Assay

Bacterial genomic DNA was extracted using a Bacterial Genomic DNA Extraction Kit (TIANGEN, Beijing, China). Approximately 400 ng of genomic DNA was mixed with the peptides (1× to 8 × MIC) for 30 min. The mixtures were subjected to agarose gel electrophoresis with a concentration of agarose of 1%. The DNA bands were observed by ultraviolet (UV) illumination with an Image Quant 300 gel documentation system (GE Healthcare, Marlborough, MA, USA).

### 2.17. In Vivo Antibiofilm Activity

Female BALB/c (18–20 g) mice were fed adaptively for one week, and then, a biofilm infection model was constructed as previously described [21]. In brief, an incision (approximately 1 cm) was made on the dorsal region of the mice, and a 1-cm sterile urinary catheter was placed in the incision. Sterile surgical threads were used to suture the incision. For bacterial preparation, *S. aureus* ATCC 25923 and MRSA ATCC 33591 were diluted to 1 × 10^6^ CFU/mL. One hundred microliters of bacterial suspension were injected into the catheter. Mice in groups of ten were injected with 100 μL of PBS as a negative control. One hundred microliters of AML (1 μg/mL for *S. aureus* and 64 μg/mL for MRSA) were set as the antibiotic control, and 100 μL of peptides (16 μg/mL) were set as the experimental group. Mice were treated twice a day for three days. Finally, the catheters were placed in 1 mL of sterile PBS. After sonication for 20 min, the number of colonies was recorded after proper dilution and plating on Mueller–Hinton agar plates.

### 2.18. Statistical Analysis

All experiments were repeated at least three times, and the statistical analysis was performed using GraphPad Prism 8.0 software (San Diego, CA, USA).

## 3. Results

### 3.1. Design and Characterization of the Peptides

The synthetic Feleucin-K3 analogs were mainly modified by α-(4-pentenyl)-Ala to improve the antimicrobial activity, stability and decrease the hemolytic activity. Sequence truncation is an effective strategy to obtain shorter peptides while maintaining the activity [22]. Moreover, the first and fourth residues, Phe and Leu, were confirmed at key positions affecting the structure and antimicrobial activity of Feleucin-K3 in our previous study [11], and we designed and synthesized some analogs containing eight amino acid residues. The masses of the peptides were characterized by ESI-MS and were shown in Table 1. The purity (>95%) and retention time (T_R_) were obtained by RP-HPLC. The T_R_ reflected the hydrophobicity, and the T_R_ values were listed from long to short (from strong hydrophobicity to weak) in the following order: K63 > K71 > K69 > K68 > K67 > K70 > K64 > K65 > K66.

**Figure 1:**
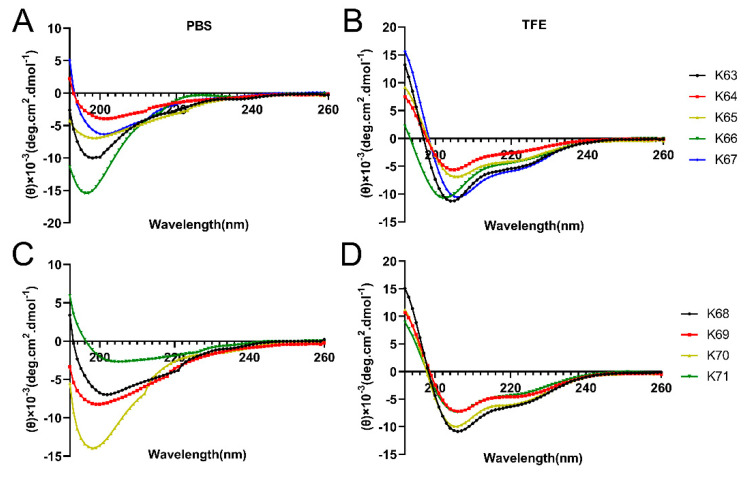
The secondary structure of Feleucin-K3 analogs in (**A**,**C**) 0.01-M PBS (pH 7.4) and (**B**,**D**) 50% TFE. The mean residue ellipticity was plotted against the wavelength.

The CD spectra were showed in Figure 1. In 0.01-M PBS, the secondary structures of the Feleucin-K3 analogs had no regularity. The α-helical content was calculated using the K2D3 algorithm (http://cbdm-01.zdv.uni-mainz.de/~andrade/k2d3/, accessed on 23 May 2020). In 50% TFE, K63, K67, K68 and K70 had increased α-helical contents compared to PBS. These analogs displayed two minimum peaks at 208 and 220 nm, which were consistent with the α-helical structure. It is worth nothing that the α-helical contents of these synthetic analogs decreased compared with Feleucin-K3, indicating that the introduced unnatural amino acid might cause a decrease in amphipathic conformation.

### 3.2. Antimicrobial Activity In Vitro

As shown in Table 2, the analogs K65, K67, K68, K69, K70 and K71, with α-(4-pentenyl)-Ala replacements, had enhanced antimicrobial activity against the standard strains, with MICs ranging from 4 to 8 µg/mL. Moreover, for MRSA and *A. baumannii* isolated from the clinic, the antimicrobial activity of these peptides was significant in comparison with the antibiotics tested (Table 3 and Table 4). The MICs of the Feleucin-K3 analogs were 4–8 µg/mL, which were much lower than the MICs of AML against MRSA (up to 128 µg/mL) [16]. It was also found in previous studies that the MICs of imipenem against multidrug-resistant *A. baumannii* were as high as 64 µg/mL, indicating that the antimicrobial activity was significantly weaker than that of the designed Feleucin-K3 analogs [16]. Notably, these Feleucin-K3 analogs had more powerful antimicrobial activities than Magainin 2, which has been studied in greater detail. The antimicrobial activity of K63, K64 and K66 containing eight residues was unsatisfactory, implying that the first residue (Phe) was critical to the structural integrity and antimicrobial activity.

### 3.3. Salt Stability

Generally, in the presence of physiological salt conditions, the antimicrobial activity is affected. Herein, the stability of the Feleucin-K3 analogs in 150-mM NaCl was determined, and the results are shown in Table 5. In 150-mM NaCl, the antimicrobial activity of K68 against all tested bacteria was not affected by monovalent ions (Na^+^), and its MICs did not increase. The MICs of the other peptides increased slightly by approximately two- to four-fold, but the activities of these analogs remained desirable. Notably, the antimicrobial activity of all analogs against MRSA ATCC 33591 was still powerful under the physiological salt conditions, with the MICs ranging from 4 to 8 µg/mL.

### 3.4. Hemolytic Activity

**Figure 2:**
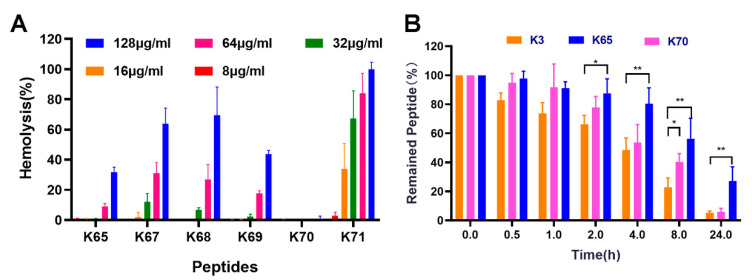
(**A**) Hemolytic activity of Feleucin-K3 analogs against mouse RBCs. The effect of 2% Triton X-100 on destroying the RBCs was regarded as a positive control and PBS as a negative control. (**B**) The serum stability of Feleucin-K3 and its analogs. The remaining peptides were determined using RP-HPLC after incubation with human serum for different times. The symbols represent the means ± standard deviations from triplicate determinations.

The hemolytic activities of the Feleucin-K3 analogs showed a significant difference compared with the parent Feleucin-K3 (Figure 2). In our previous study, Feleucin-K3 showed toxicities at high concentrations. The hemolytic rate was more than 40% at the concentration of 256 µg/mL [11]. K65 and K70, the analogs with α-(4-pentenyl)-Ala at the N-terminus and C-terminus, respectively, showed low hemolytic activity. Notably, the hemolysis rates of K65 were 9.08% and 31.6% at concentrations of 64 µg/mL and 128 µg/mL, respectively. The hemolysis rates of K70 were less than 1%, even at the maximum concentration (128 µg/mL). The hemolytic activity of K70 decreased significantly compared with Feleucin-K3. The other analogs showed negligible hemolytic activity at their antimicrobial concentrations but, unfortunately, showed high hemolysis at high concentrations.

### 3.5. Serum Stability

Human serum digests realistically imitate the actual situation in organisms. Herein, the stabilities were evaluated for the Feleucin-K3 analogs by determining the amounts of remaining peptides after different incubation times. As shown in Figure 2, approximately 80% of Feleucin-K3 was degraded after incubation with the human serum for 8 h. After 24 h, the content of Feleucin-K3 was only 5.83%, implying that it was completely degraded. In contrast, the retention rate of K65 was 56.4% and 27.3% after 8 h and 24 h, respectively. K70 had different levels of degradation after incubation for different times. The statistically significant differences could be determined compared with Feleucin-K3 when they were incubated with the serum for 8 h. The serum stabilities of K65 showed statistically significant differences compared with that of Feleucin-K3 at 2 h, 4 h, 8 h and 24 h.

### 3.6. Time-Kill Kinetic Curves

**Figure 3:**
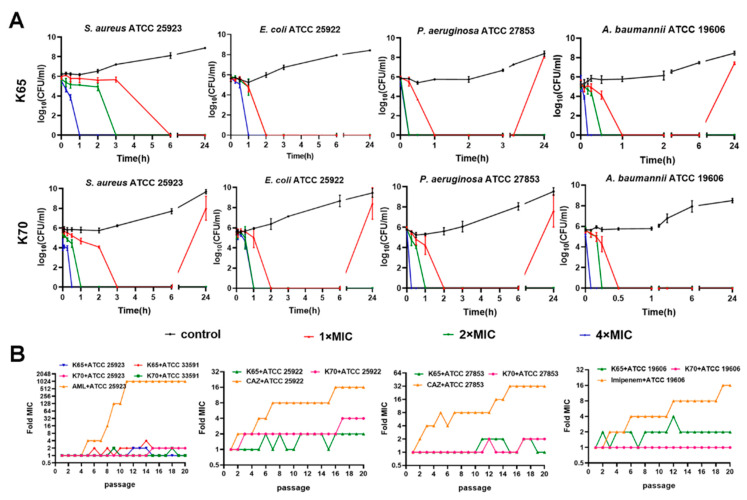
(**A**) The time-kill kinetics of K65 and K70 against *S. aureus* ATCC 25923, *E. coli* ATCC 25922, *P. aeruginosa* ATCC 27853 and *A. baumannii* ATCC 19606. (**B**) Resistance induced by K65 and K70 by the sequential passaging method. The fold changes of the MICs of AML, CAZ and Imipenem were also recorded to detect the resistance.

K65 and K70 were chosen to test their bactericidal activity after different treatment times. In our study, the bactericidal effects of K65 and K70 were rapid and related to both the time and concentration (Figure 3). For *S. aureus* and *E. coli*, K70 (4 × MIC) killed the bacteria in 30 min or 1 h, which was comparable to K65. However, K65 at high concentrations had a more powerful short-term killing effect against *P. aeruginosa* than K70. *P. aeruginosa* could not be detected after 15 min of treatment with K65. Moreover, the bactericidal activity of K70 against *A. baumannii* was extremely significant. No bacteria were detected after treatment for 5 min with 4 × MIC of K70.

### 3.7. Resistance Development Assay

The sequential passaging method was used to determine the bacterial resistance development induced by K65 and K70. As shown in Figure 3, the MICs of K65 and K70 against the standard strains (including *S. aureus*, *A. baumannii*, *E. coli* and *P. aeruginosa*) and drug-resistant MRSA displayed only slight fluctuations, showing a one- to four-fold increase after 20 passages. In sharp contrast, the antibiotic resistance increased dramatically. AML-resistant mutants were produced as early as passage 5, and the MICs increased by a maximum of 1024-fold against *S. aureus*. The MICs with CAZ increased 32-fold and 16-fold against *P. aeruginosa* and *E. coli*, respectively. In addition, the MICs of imipenem against *A. baumannii* increased by a maximum of 16-fold. These results indicated that K65 and K70 showed significant advantages by posing as a low risk for resistance development in comparison with the antibiotics tested.

### 3.8. Biofilm Inhibition Activity In Vitro

**Figure 4:**
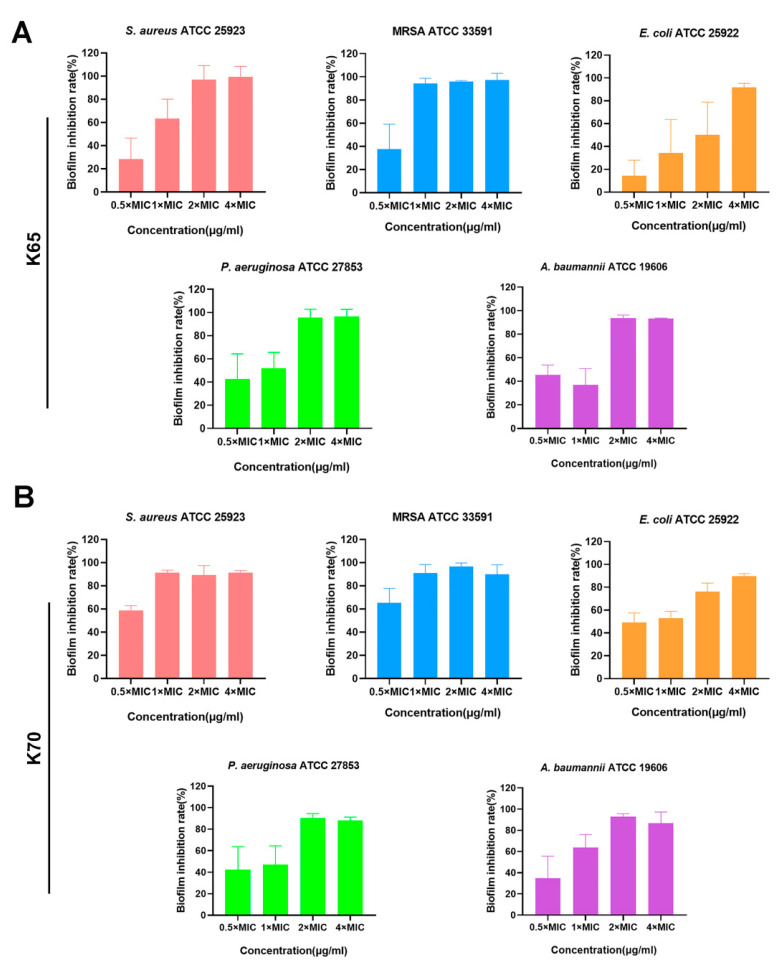
Biofilm inhibitory activity of (**A**) K65 and (**B**) K70 against *S. aureus* ATCC 25923, MRSA ATCC 33591, *E. coli* ATCC 25922, *P. aeruginosa* ATCC 27853 and *A. baumannii* ATCC 19606.

**Figure 5:**
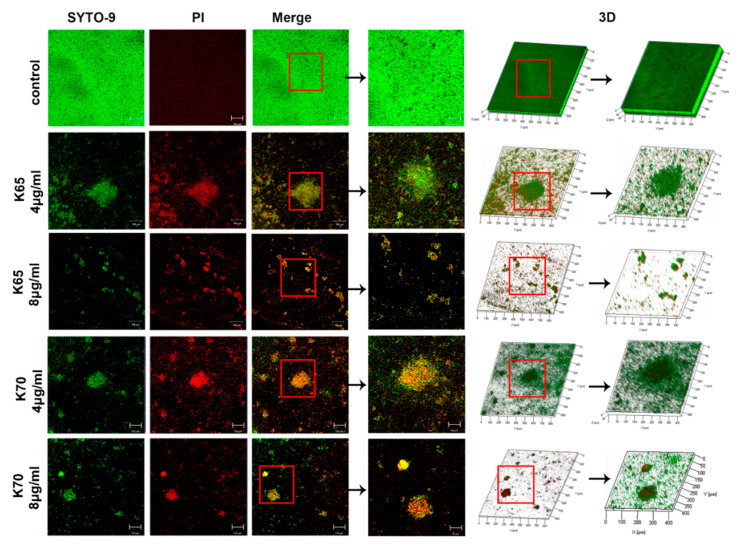
Biofilm inhibitory activity of K65 and K70 against MRSA ATCC 33591 was determined by CLSM using a LIVE/DEAD Biofilm Viability Kit. Bacteria were incubated with these analogs (4 μg/mL and 8 μg/mL) for 24 h to form the biofilm. The mixed dyes of PI and SYTO9 (6 μg/mL) were used to stain biofilm dead and live cells. The three-dimensional structures were imaged by the Z-stack mode of the CLSM.

**Figure 6:**
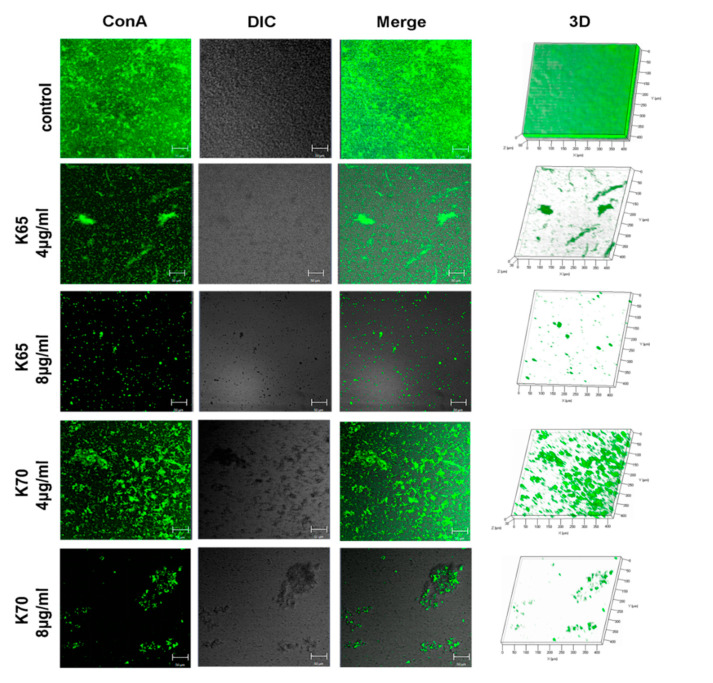
The biofilm inhibitory activity of K65 and K70 against MRSA ATCC 33591 was determined by CLSM using concanavalin A conjugates. The bacteria were incubated with these analogs (4 μg/mL and 8 μg/mL) for 24 h to form the biofilm. Concanavalin A conjugates (40 μg/mL) were used to stain the exopolysaccharide matrix. The three-dimensional structures were imaged by the Z-stack mode of the CLSM.

**Figure 7:**
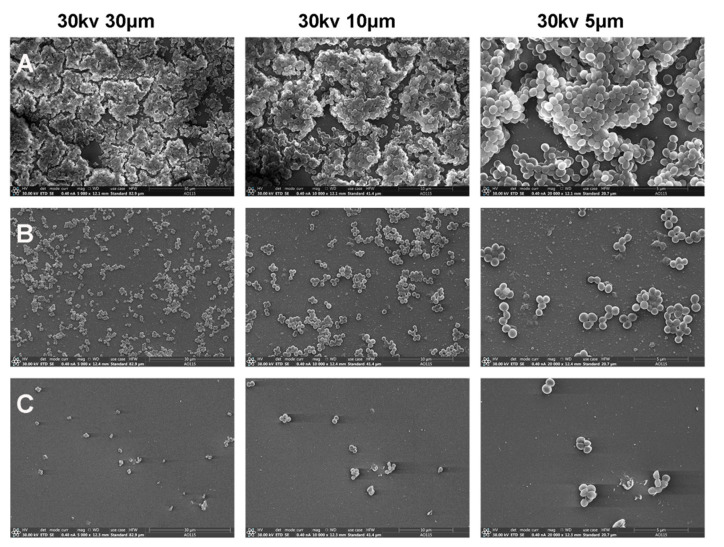
Impact of K70 on the biofilm of MRSA ATCC 33591, as determined by SEM. (**A**) Untreated biofilm cells, (**B**) biofilm cells treated with K70 at 4 μg/mL and (**C**) biofilm cells treated with K70 at 8 μg/mL.

The antibiofilm activities of the peptides against both sensitive bacteria and MRSA were evaluated, and the results are shown in Figure 4 and Table 6. K65 and K70 (0.5 × to 4 × MIC) significantly inhibited the bacterial biofilm formation. Notably, both K65 and K70 displayed perfect MRSA biofilm inhibition activity at the low concentration of 8 µg/mL. Therefore, their antibiofilm effects against MRSA were further explored using CLSM (Figure 5 and Figure 6) and SEM (Figure 7).

#### 3.8.1. CLSM

The ability of K65 and K70 to inhibit the MRSA biofilm formation was assessed by CLSM. As shown in Figure 5, almost all MRSA biofilm cells without peptide treatment were stained green by SYTO9, implying that mature and thick biofilms developed in the control group. However, K65 and K70 significantly inhibited the MRSA biofilm formation. In the low-concentration groups, there were numerous dead biofilm cells. In the high-concentration groups, only sporadic fluorescence was observed in the field of vision. In the 3D images, the thicknesses of the biofilms exposed to K65 (40 µm at 4 µg/m and 10 µm at 8 µg/mL) and K70 (30 µm at 4 µg/m and 20 µm at 8 µg/mL) decreased tremendously compared with the untreated biofilm (60 µm).

In addition, exopolysaccharides are one of the main extracellular polymeric substance (EPS) components, which are beneficial for maintaining a mature biofilm structure. In our study, K65 and K70 showed a strong ability to inhibit the formation and accumulation of exopolysaccharides (Figure 6). The 3D images showed that the thicknesses of the biofilms exposed to K65 (30 µm at 4 µg/mL and 20 µm at 8 µg/mL) or K70 (30 µm at 4 µg/mL and 20 µm at 8 µg/mL) decreased significantly in comparison with the control group (80 µm).

#### 3.8.2. SEM

K70 was chosen to observe its antibiofilm effects on MRSA using SEM. As shown in Figure 7, in the control group, the bacteria adhered to form mature biofilms characterized by structured communities instead of individual colonies (Figure 7A). In the low-concentration group (4 µg/mL), the number of biofilm cells were significantly reduced (Figure 7B). In the high-concentration group (8 µg/mL), the bacteria appeared sporadically and were not enough to form biofilms (Figure 7C). These results indicated that the inhibitory effects of K70 on MRSA biofilm formation were remarkable and concentration-dependent.

### 3.9. Membrane Mechanism of Action of Feleucin-K3 Analogs

**Figure 8:**
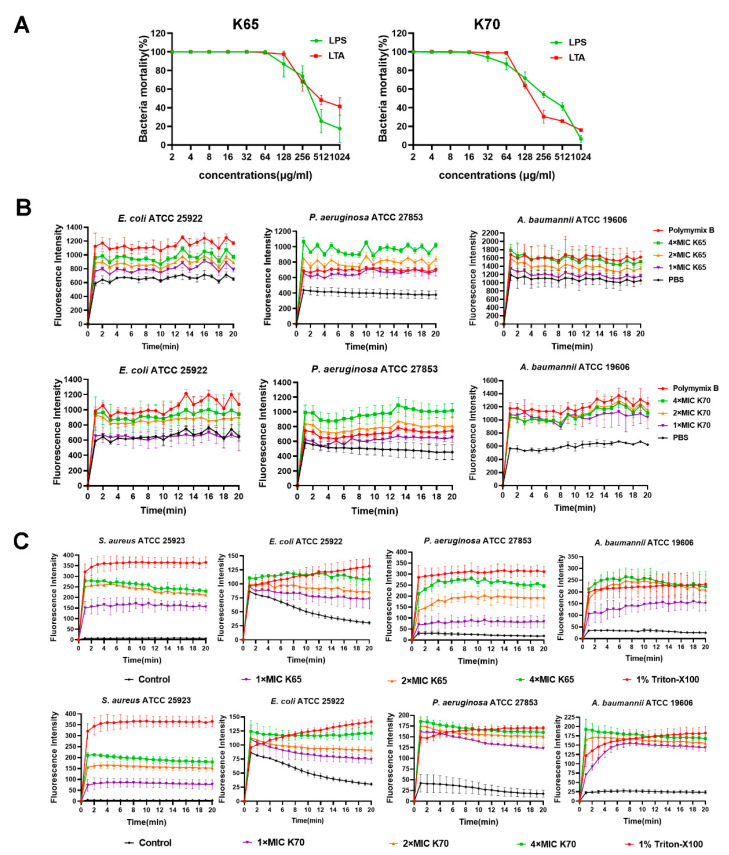
(**A**) Bacteria mortality of *S. aureus* ATCC 25923 and *E. coli* ATCC 25922 as a function of the LPS/LTA concentrations. (**B**) The outer membrane permeabilization of *E. coli* ATCC 25922, *P. aeruginosa* ATCC 27853 and *A. baumannii* ATCC 19606 induced by different concentrations of K65 and K70. (**C**) The cytoplasmic membrane depolarization of *S. aureus* ATCC 25923, *E. coli* ATCC 25922, *P. aeruginosa* ATCC 27853 and *A. baumannii* ATCC 19606 induced by different concentrations of K65 and K70.

In this study, the interactions between the Feleucin-K3 analogs and the cell membrane components were successively investigated to clarify the mode of membrane action of the Feleucin-K3 analogs. In Figure 8A, the coincubation of LPS/LTA with K65 and K70 had a concentration-dependent inhibitory effect. The antimicrobial activity could be completely inhibited when these peptides were bound with high concentrations of LPS/LTA.

Then, after the bacterial treatment with different concentrations of analogs, the significantly increased fluorescence of NPN implied a strong damaging effect to the bacterial outer membrane (Figure 8B). Notably, the level of permeabilization induced by high concentrations of K65 and K70 was stronger than that induced by low concentrations.

Cytoplasmic membrane depolarization was detected using DiSC3(5). As shown in Figure 8C, the fluorescence intensity increased rapidly, indicating that the membrane potential dissipated. However, the cytoplasmic membrane depolarization of Gram-negative bacteria induced by the analogs was stronger than that of Gram-positive *S. aureus*. For *E. coli* and *A. baumannii*, the effects of K65 were similar to those of K70. For *P. aeruginosa*, K65 at high concentrations showed substantially stronger cytoplasmic membrane depolarization than K65 at low concentrations.

**Figure 9:**
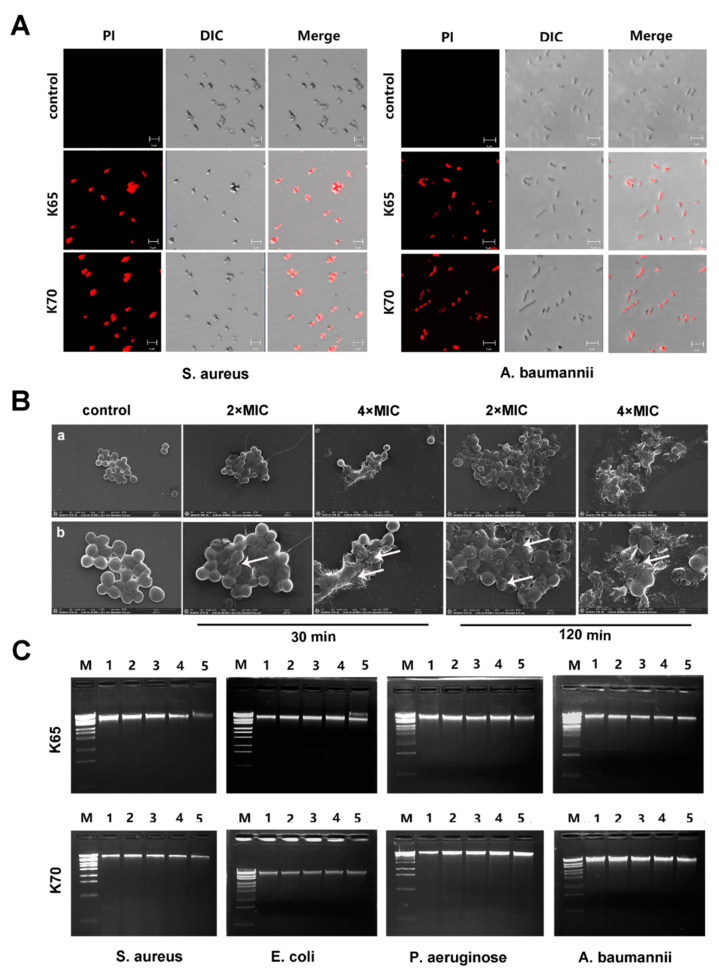
(**A**) PI uptake of *S. aureus* ATCC 25923 and *A. baumannii* ATCC 19606 treated with K65 and K70 at 4 × MIC for 30 min. (**B**) SEM micrographs of MRSA ATCC 33591 treated with K70 at 2 × MIC or 4 × MIC for 30 min or 120 min. Arrows indicate that the membrane displayed a rough and irregular atrophy. (**a**) The magnification is 30,000×. (**b**) The magnification is 60,000×. (**C**) The interaction of K65 and K70 with bacterial genomic DNA. The lanes represented the following: lane M, DNA marker (λ-EcoT4 I digest); lane 1, DNA only; lane 2, 1 × MIC; lane 3, 2 × MIC; lane 4, 4 × MIC and lane 5, 8 × MIC.

The PI uptake abilities of *S. aureus* and *A. baumannii* treated with analogs K65 and K70 were analyzed, and the results are shown in Figure 9A. The bacteria treated with analogs K65 and K70 presented significant red signals, implying strong membrane destruction.

Additionally, the membrane-damaging effects of K70 on MRSA were observed using SEM. As shown in Figure 9B, the surface of the cell membrane was rough, shrunken and wrinkled when the MRSA cells were exposed to K70 for 30 min. After 120 min, severe membrane damage, such as membrane surface collapse and the leakage of intracellular components, was observed. In addition, K70 at a high concentration (4 × MIC) had a more severe damaging effect than that at a low concentration (2 × MIC), indicating that the damaging effects of K70 on the MRSA membrane were time- and concentration-dependent.

### 3.10. DNA-Binding Affinity

A DNA-binding assay was used to explore whether the Feleucin-K3 analogs interacted with bacterial genomic DNA. As shown in Figure 9C, both K65 and K70, ranging from 1 × MIC to 8 × MIC, did not hinder the migration of genomic DNA within the agarose gels, indicating that these analogs did not kill the bacteria by combining with genomic DNA.

### 3.11. Antibiofilm Activity In Vivo

**Figure 10:**
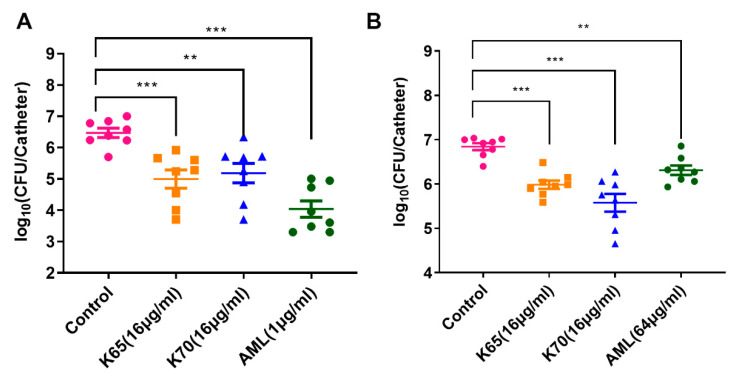
Antibiofilm activity of K65 and K70 in vivo. Catheter biofilm infections caused by *S. aureus* or MRSA were established. Animals were treated with 100 μL of PBS, AML, K65 and K70 injected into the catheter twice a day. After 3 days, the bacterial load in the catheter was calculated. (**A**) The inhibition effect of the *S. aureus* ATCC 25923 biofilm. (**B**) The inhibition effect of the MRSA ATCC 33591 biofilm. The experiments were repeated three times, and the results were consistent. The data represent the mean ± SD. Statistically significant differences were noted with an asterisk (** 0.001 < *p* < 0.01 and *** *p* < 0.001).

In this experiment, K65 and K70 were chosen to determine their ability to treat biofilm-associated infections in mice. *S. aureus* and MRSA were used as the experimental strains. As shown in Figure 10A, the effects of K65 and K70 (16 µg/mL) on *S. aureus* biofilm inhibition was remarkable. However, compared with the antibiotic AML (1 µg/mL), the inhibitory effects of the peptides were not as potent as that of AML. As shown in Figure 10B, 16 µg/mL of K65 and K70 were used as the experimental group. After the treatment for 3 days, the number of MRSA ATCC 33591 cells in each experimental group was significantly reduced. Notably, compared with 64 µg/mL of AML, K65 and K70 inhibited the formation of the MRSA biofilms with higher potency. These results indicated that K65 and K70 had potent biofilm inhibition effects. More importantly, there were remarkable advantages for these analogs compared with antibiotics in the treatment of biofilm infections caused by multidrug-resistant bacteria.

## 4. Discussion

The introduction of unnatural amino acids may cause major functional differences in AMPs, which, in turn, affects their ability to kill microorganisms and can induce toxicity to host cells [23]. α-(4-Pentenyl)-Ala is an unnatural hydrophobic amino acid that affects the hydrophobicity and amphipathicity of entire peptides. Herein, α-(4-pentenyl)-Ala was introduced into the short nonapeptide Feleucin-K3. The antimicrobial activity and structure–activity relationships of the synthetic analogs were studied to search for excellent antibacterial lead compounds.

The T_R_ values obtained from RP-HPLC reflect the hydrophobicity. AMPs with greater hydrophobicity generally showed an increase in T_R_ [24]. Among these analogs, the analogs with unnatural hydrophobic amino acid replacements had enhanced hydrophobicity and antimicrobial activity. K63 had the highest hydrophobicity but the worst antimicrobial activity among the analogs. The results displayed a U-shaped relationship between the activity of the analogs and their hydrophobicity [25]. Increasing the hydrophobicity could enhance the antimicrobial activity until an optimal hydrophobicity threshold was reached [26]. This is consistent with the known antimicrobial mechanism. AMPs with higher hydrophobicity can be inserted into a hydrophobic core, resulting in the disruption of the membrane structure or cell lysis [3]. AMPs with a hydrophobicity that is too high might self-aggregate, resulting in a decrease in antimicrobial activity [27]. In addition, excessive hydrophobicity was highly correlated with hemolytic activity. In this study, K71 had greatly enhanced hydrophobicity. However, this excessive hydrophobicity might be a reason why its hemolytic activity was significantly higher than that of the other analogs. Compared with the other analogs, K65 and K70 had enhanced hydrophobicity and significantly reduced hemolytic activity.

AMP amphipathicity has been considered a feature that directly affects antimicrobial and hemolytic activity [28]. In this experiment, these analogs with α-(4-pentenyl)-Ala substitution had decreased α-helical contents and enhanced antimicrobial activity compared with Feleucin-K3. However, they showed a significant difference in hemolytic activity. The influence of α-(4-pentenyl)-Ala substitution on hemolytic activity was associated with the α-helical content and the position of this substitution [29]. The α-helical contents of the Feleucin-K3 analogs were lower than that of Feleucin-K3, implying that the introduction of this unnatural amino acid led to a decrease in amphipathicity. Imperfect amphiphilicity may contribute to a reduction in hemolytic activity [30]. Furthermore, it was worth noting that analogs in which substitutions occurred at the N-terminus (K65) and C-terminus (K70) had lower hemolytic activity than analogs where the substitutions occurred near the center of the sequence. In particular, it was found that the C-terminal residue (leucine) exchange with α-(4-pentenyl)-Ala not only enhanced the antimicrobial activity but also strongly impaired the hemolytic activity. This might be because K70 had an excellent hydrophobic/hydrophilic balance, which was optimal for peptide selectivity to the cell and interactions with the membrane core penetrating into the cell [31].

Many reports showed that peptides with reasonably modulated α-helical structures displayed great selectivity against prokaryotic and eukaryotic cells [19]. In our previous study, K59 was an analog of the α-(4-pentenyl)-Ala substitution that took place in the fourth position [12]. K70 exhibited significant antimicrobial activity without hemolytic activity, whereas K59 showed nonnegligible hemolytic activity at high concentrations. We guessed that decreasing the α-helical content (15.62%) of K70 might cause lower hemolytic activity compared with K59 (41.93%). Moreover, the introduction of unnatural amino acid α-(4-pentenyl)-Ala was beneficial for the resistance against proteases, regardless of whether the substitution occurred in terminus (K65 and K70) and near the center of the sequence (K59).

Cations in physiological salt environments, such as Na^+^, hamper electrostatic interactions, resulting in the resistance of AMPs in high-salt environments [32,33]. Moreover, natural AMPs are susceptible to degradation by proteases or a combination with serum albumin, resulting in impaired antimicrobial activity [34]. Modifying natural amino acids can increase their resistance to proteolytic degradation and circumvent their susceptibility [7]. In line with our results, the Feleucin-K3 analogs maintained their antimicrobial activity, especially against MRSA. These peptides had enhanced stability compared with Feleucin-K3 in the presence of human serum (Figure 2). These analogs showed low α-helical contents but displayed an enhanced β-strand content in comparison with Feleucin-K3 (Table S1). Therefore, we speculated that this was possibly because α-(4-pentenyl)-Ala, an alanine analog with a long side chain alkene, was introduced to Feleucin-K3. This special, long hydrophobic alkene side chain substituent not only affected the hydrophobicity but also considerably affected the secondary structure. The hydrophobicity and secondary structure are closely intertwined in regard to the stability of AMPs.

Frog skin is the most abundant source of AMPs, such as Magainin, Temporin and Esculentin, that have been found and widely reported [35,36]. Magainin 2 is a membrane-active AMP that was isolated from the skin of the African clawed frog. It was confirmed that this AMP can kill bacteria via electrostatic attractions and form a pore but cannot retard the migration of DNA [37]. The mode of action was similar to that of the Fekeucin-K3 analogs tested in this study. However, the new synthetic Feleucin-K3 analogs had more effective antimicrobial activity than Magainin 2. In a recent study, α-(4-pentenyl)-Ala was reported being used for modifying Magainin 2. The introduction of α-(4-pentenyl)-Ala in the *i/i*+ 4 position could stabilize its helical structure and improve the bioactivity [38]. In this study, although introducing the α-(4-Pentenyl)-Ala with a hydrocarbon side chain decreased the α-helical content, it also enhanced the antimicrobial activity and reduced the toxicity.

Biofilms can be simply defined as a structured community characterized by cells that are encased in a matrix of self-produced EPSs [39]. Bacterial biofilms present a high resistance to antibiotics, and their antibacterial tolerance is 10- to 1000-fold higher than that of planktonic bacteria. Costerton et al. pointed out that an inherent biofilm resistance to antibiotics is the main reason for many persistent and chronic bacterial infections [40]. In our study, K65 and K70 showed significant antibiofilm activity in vitro/in vivo. Additionally, the antibiofilm activity of these analogs against MRSA at low concentrations was better than the effects of AML on MRSA at high concentrations in biofilm-associated infections. We speculated that the rapid killing ability of these peptides was crucial, which extremely reduced the adhesion of the biofilm bacteria. Moreover, K65 and K70 significantly inhibited the formation of the exopolysaccharides associated with biofilm maturation, thereby reducing the development of bacterial resistance by means of antibiofilms [41].

For the peptide–membrane interaction, LPS and LTA, as the main anionic polymers in the bacterial membrane, were confirmed to bind with high affinity to the positively charged analogs. These polymers might act to trap the AMPs, facilitating the penetration of the cell wall [42]. Subsequently, the outer membrane permeability and cytoplasmic membrane depolarization induced by K65 and K70 rapidly increased and had significant time- and concentration-dependent effects, indicating that the peptide–membrane interaction might be related to channel or pore formation, thus leading to cellular component leakage and cell death [43]. Interestingly, depolarization of the *S. aureus* cytoplasmic membrane induced by the Feleucin-K3 analogs was weaker than that in Gram-negative bacteria. An explanation for this result might be the structural differences between the bacterial cell walls. AMPs competitively displace the divalent ions (Mg^2+^) and calcium ions (Ca^2+^) of the outer membrane by penetrating both the outer and cytoplasmic membranes in Gram-negative bacteria [44]. For Gram-positive bacteria, AMPs can cross peptidoglycan and other components before depolarization of the cytoplasmic membrane [45]. The PI uptake and SEM assays also supported that analogs K65 and K70 displayed antimicrobial activity mainly through membrane disruption mechanisms.

## 5. Conclusions

In our study, a series of (4-pentenyl)-Ala-substituted Feleucin-K3 analogs were designed and synthesized. Among the synthetic analogs, we successfully screened K65 and K70, whose N-terminal and C-terminal residues were substituted by (4-pentenyl)-Ala, respectively, which showed improved antimicrobial activity, stability, low hemolytic activity and AMR. In particular, K70 had an excellent hydrophobic/hydrophilic balance, producing no hemolytic activity at high concentrations. Considering the role of biofilms in AMR, the antibiofilm activity of K65 and K70 was estimated. Our results indicated that these compounds effectively inhibited biofilm formation in vitro/in vivo and that the inhibitory activity against MRSA was better than that of traditional antibiotics. In addition, K65 and K70 exhibited a strong membrane destabilization ability and damage to the membrane integrity, leading to bacterial death. In summary, these results demonstrated that the introduction of the unnatural amino acid α-(4-pentenyl)-Ala was a potential strategy in developing potent antimicrobial agents.

## Figures and Tables

**Table 1:** The main physicochemical properties of the Feleucin-K3 analogs.

Peptides.	Sequence ^a^	Mass (Da) ^b^	Number of Amino Acids	Net Charge	T_R_ (Min) ^c^	α-Helix (%) PBS ^d^	α-Helix (%) TFE ^d^	Purity (%) ^c^
Feleucin-K63	LKLLKKLL-NH_2_	967.7	8	+4	23.738	4.600	14.970	96.070
Feleucin-K64	LKα-(4-pentenyl)-AlaLKKLL-NH_2_	993.8	8	+4	17.768	3.330	2.900	100.000
Feleucin-K65	α-(4-pentenyl)-AlaLKLLKKLL-NH_2_	1106.8	9	+4	17.762	2.920	5.120	98.900
Feleucin-K66	α-(4-pentenyl)-AlaLKAKKLL-NH_2_	951.7	8	+4	14.450	3.140	5.050	96.250
Feleucin-K67	Fα-(4-pentenyl)-AlaKLLKKLL-NH_2_	1140.8	9	+4	18.086	3.620	18.610	98.450
Feleucin-K68	FLKLα-(4-pentenyl)-AlaKKLL-NH_2_	1140.8	9	+4	18.466	2.930	19.640	100.000
Feleucin-K69	FLKLLKKα-(4-pentenyl)-AlaL-NH_2_	1140.8	9	+4	18.635	2.990	7.140	100.000
Feleucin-K70	FLKLLKKLα-(4-pentenyl)-Ala-NH_2_	1140.8	9	+4	17.942	3.070	15.620	100.000
Feleucin-K71	FLKLLα-(4-pentenyl)-AlaKLL-NH_2_	1125.8	9	+3	20.493	1.870	5.240	100.000

^a^ Red indicates the substitution site. ^b^ Mass was measured by ESI-MS. ^c^ T_R_ (min) and purity were measured by RP-HPLC. ^d^ α-helix (%) was measured by CD spectroscopy.

**Table 2:** MICs of the Feleucin-K3 analogs against the standard strains.

Peptides	MIC (μg/mL)			
	*E. coli* ATCC 25922	*S. aureus* ATCC 25923	*P. aeruginosa* ATCC 27853	*A. baumannii* ATCC 19606
Feleucin-K63	>128	>128	64	>128
Feleucin-K64	16	16	4	64
Feleucin-K65	8	8	8	4
Feleucin-K66	>128	>128	>128	>128
Feleucin-K67	8	4	8	4
Feleucin-K68	8	4	8	4
Feleucin-K69	8	4	8	8
Feleucin-K70	8	8	8	8
Feleucin-K71 Magainin 2	16 64	4 >256	8 256	4 16

**Table 3:** MICs of the Feleucin-K3 and analogs against the multidrug-resistant *S. aureus*.

MIC (μg/mL)							
MRSA	K3	K65	K67	K68	K69	K70	K71
MRSA ATCC 33591	8	4	4	4	4	8	4
MRSA 48	8	4	4	4	4	8	4
MRSA 54	8	4	4	4	4	8	4
MRSA 936	8	4	4	4	4	8	4
MRSA 52	8	4	4	8	4	8	4
MRSA 74	8	4	4	4	8	8	4
MRSA 23	8	4	4	4	4	8	4
MRSA 75	8	4	4	4	4	8	4
MRSA 113	8	8	4	4	4	8	4
MRSA 51	8	8	4	4	4	8	4
MRSA 71	8	4	4	8	4	8	4
*S. aureus* 794	16	8	8	8	8	8	16
*S. aureus* 725	8	4	4	4	4	8	4

**Table 4:** MICs of the Feleucin-K3 and analogs against the multidrug-resistant *A. baumannii*.

MIC (μg/mL)							
*A. baumannii*	K3	K65	K67	K68	K69	K70	K71
*A. baumannii* 9828	16	4	4	4	8	8	16
*A. baumannii* 9840	8	4	4	4	8	8	8
*A. baumannii* 9896	8	4	4	4	8	4	8
*A. baumannii* 91152	8	4	4	4	8	8	16
*A. baumannii* 98110	8	4	4	4	8	8	16
*A. baumannii* 92359	8	4	4	4	4	8	16
*A. baumannii* 97830	8	4	4	4	8	8	32
*A. baumannii* 9234	8	4	4	4	8	8	8
*A. baumannii* 5444	8	4	4	4	8	8	8
*A. baumannii* 9236	16	4	4	4	8	8	16
*A. baumannii* 91869	8	4	4	4	8	8	8
*A. baumannii* 91199	8	4	4	4	8	8	16
*A. baumannii* 9336	8	4	4	4	8	8	8
*A. baumannii* 91810	8	4	4	4	8	8	16
*A. baumannii* 822144	8	8	4	4	8	8	16
*A. baumannii* 91944	8	4	4	4	8	8	16
*A. baumannii* 91105	8	4	4	4	8	4	8
*A. baumannii* 51243	8	4	4	4	8	8	32
*A. baumannii* 8309	8	4	4	4	8	8	8
*A. baumannii* 9331	16	8	8	8	8	16	64

**Table 5:** Salt stability of the Feleucin-K3 analogs.

Peptides	*E. coli* ATCC 25922		*S. aureus* ATCC 25923		*P. aeruginosa* ATCC 27853		*A. baumannii* ATCC 19606		MRSA ATCC 33591	
	150-mM NaCl	Fold Change	150-mM NaCl	Fold Change	150-mM NaCl	Fold Change	150-mM NaCl	Fold Change	150-mM NaCl	Fold Change
K65	32	4	8	2	32	4	16	4	4	1
K67	16	2	4	1	8	1	8	2	4	1
K68	8	1	4	1	8	1	4	1	4	1
K69	16	2	8	2	16	2	16	2	8	2
K70	32	4	16	2	8	1	16	2	8	1
K71	16	1	4	1	16	2	8	2	4	1

**Table 6:** Antibiofilm activity.

Strains	K65 (μg/mL)			K70 (μg/mL)		
	MIC	MBIC_50_	MBIC_90_	MIC	MBIC_50_	MBIC_90_
*S. aureus* ATCC 25923	8	8	16	8	4	8
MRSA ATCC 33591	4	8	8	8	4	8
*E. coli* ATCC 25922	8	16	32	8	8	32
*A. baumannii* ATCC 19606	4	8	8	8	8	16
*P.aeruginosa* ATCC 27853	8	8	16	8	8	16

## Data Availability

Not applicable.

## Supplementary Materials

The following are available online at https://www.mdpi.com/article/10.3390/biom11050761/s1: Table S1: Circular dichroism spectrum date of Feleucin-K3 and its analogs in PBS and 50% TFE.
