# Supplementary material for: Potent Antimicrobial and Antibiofilm Activities of Feleucin-K3 Analogs Modified by α-(4-Pentenyl)-Ala against Multidrug-Resistant Bacteria
Source: Biomolecules. 2021 May 19;11(5):761. doi: 10.3390/biom11050761 (PMC8160793; doi:10.3390/biom11050761)
Supplement: Supplementary file 1 [file biomolecules-11-00761-s001.zip › biomolecules-1215188-supplementary.pdf]

Supplementary Material

Table S1. Circular dichroism spectrum date of Feleucin-K3 and its analogs in PBS and 50% TFE.

Peptide (% content[a])	PBS	50%TFE
	β-strand	β-strand
Feleucin-K3	1.95	0.46
K63	10.51	16.41
K64	14.63	23.24
K65	11.12	23.56
K66	9.71	10.93
K67	12.82	18.86
K68	12.30	17.34
K69	10.28	24.08
K70	9.38	18.09
K71	20.20	21.99

[a]The α-helix and β-strand contents of the Feleucin-K3 analogs were estimated online by K2D3 tools.

2. Quality Data for Major Synthetic Peptides Used in This Study

2.1. MS and HPLC data for Feleucin-K63

MS: expected mass: 968.3; Measured average mass: 967.7

Generic Display Report

**Analysis Info**		Acquisition Date	4/17/2019 10:45:25 AM
Analysis Name	D:\Data\201904\20190416raojing01.d		
Method	POS_TuneLow_NaTFACal_100-1200.m	Operator	BDAL@DE
Sample Name	< No Sample >	Instrument	maXis 4G
Comment			

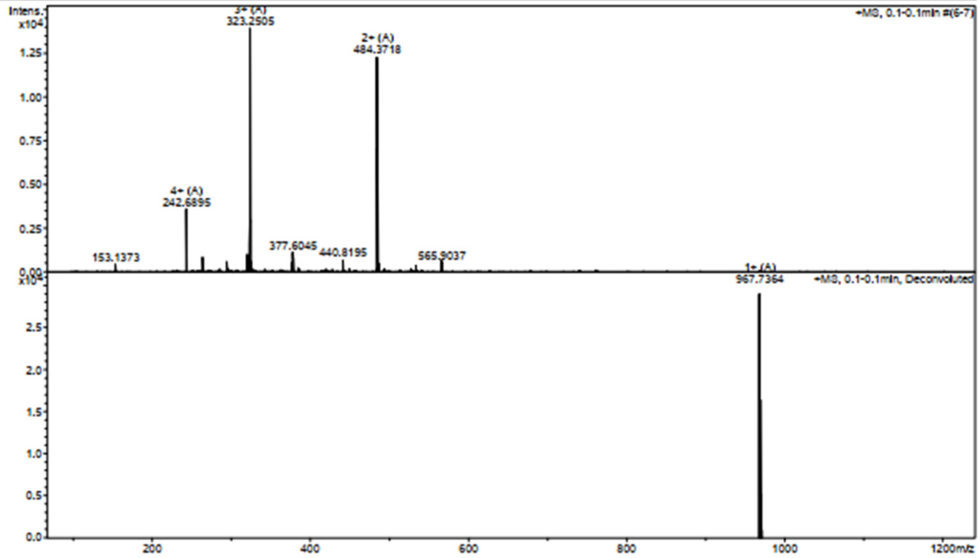

Bruker Compass DataAnalysis 4.0 printed: 4/18/2019 9:02:57 AM Page 1 of 1

Customer Peptide HPLC Report Purity: 96.07%

Sample Information

Name:	K63	Collector:	System
Type:	NO	Acquisition time:	2019-5-31 10:19:13 CST
Bottle number:	1	Method group:	0
Injection times:	1	Processing date:	2019-5-31 20:49:18 CST
Injection volume:	20.00 ul	Processing method:	0
Operation time:	30.0 Minutes	Channel name:	220.0 nm
		Channel annotation:	PDA 220.0 nm

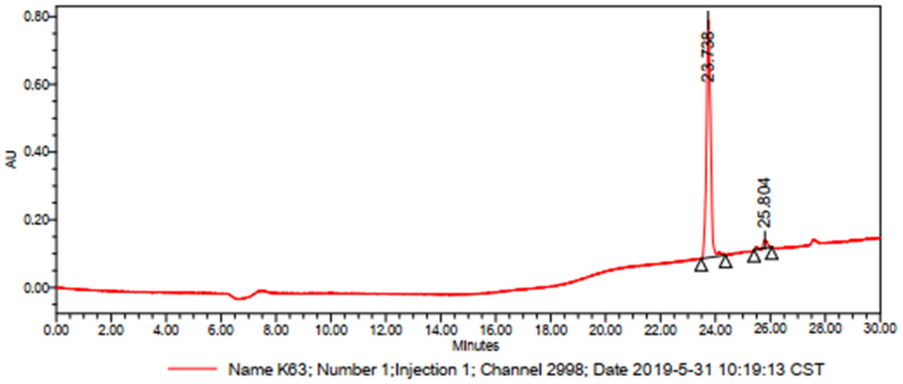

Channel: PDA 220.0 nm

	channel	retention time(min)	area	% area	peak height
1	PDA 220.0 nm	23.738	7339853	96.07	700906
2	PDA 220.0 nm	25.804	300423	3.93	23054

2.2. MS and HPLC data for Feleucin-K64

MS: expected mass: 994.5; Measured average mass: 993.8

Generic Display Report

**Analysis Info**		Acquisition Date	4/17/2019 10:46:41 AM
Analysis Name	D:\Data\201904\20190416raojing02.d		
Method	POS_TuneLow_NaTFACal_100-1200.m	Operator	BDAL@DE
Sample Name	< No Sample >	Instrument	maXis 4G
Comment			

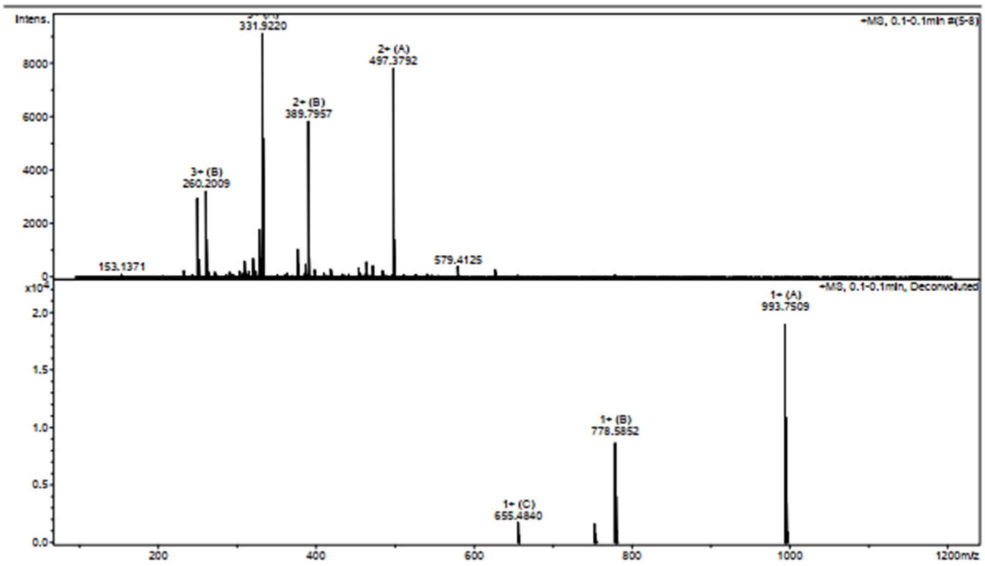

Bruker Compass DataAnalysis 4.0 printed: 4/18/2019 9:03:13 AM Page 1 of 1

Customer Peptide HPLC Report Purity: 100.00%

Sample Information

Name:	K64	Collector:	System
Type:	NO	Acquisition time:	2019-7-19 17:04:32 CST
Bottle number:	1	Method group:	peptide
Injection times:	1	Processing date:	2019-7-19 20:37:23 CST
Injection volume:	20.00 ul	Processing method:	0
Operation time:	30.0 Minutes	Channel name:	220.0 nm
		Channel annotation:	PDA 220.0 nm

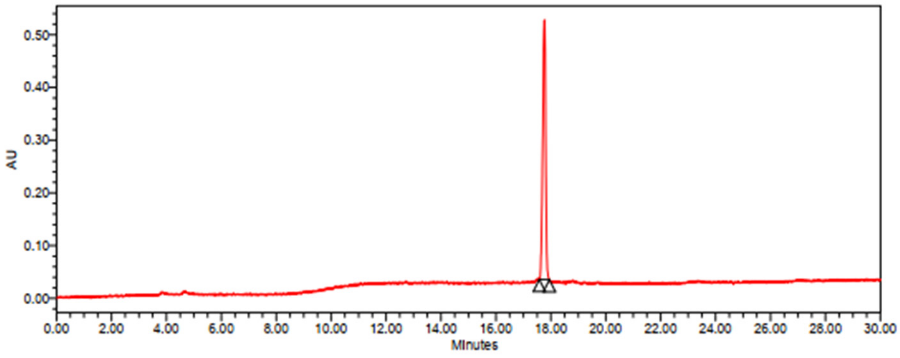

Channel: PDA 220.0 nm

	channel	retention time(min)	area	% area	peak height
1	PDA 220.0 nm	17.768	3728537	100.00	492610

2.3. MS and HPLC data for Feleucin-K65

MS: expected mass: 1107.5; Measured average mass: 1106.8

Generic Display Report

Analysis Info

Analysis Name	D:\Data\201904\20190424raojing01.d	Acquisition Date	4/25/2019 3:57:45 PM
Method	POS_TuneLow_NaTFACal_100-1200.m	Operator	BDAL@DE
Sample Name	< No Sample >	Instrument	maXis 4G
Comment			

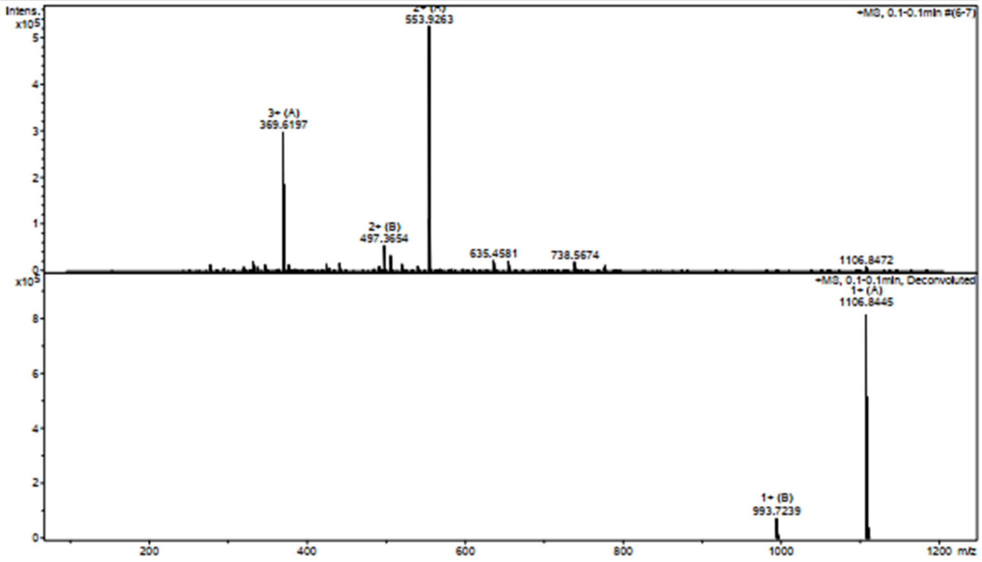

Bruker Compass DataAnalysis 4.0 printed: 4/25/2019 6:22:35 PM Page 1 of 1

Customer Peptide HPLC Report Purity:98.90%

Sample Information

Name:	K65	Collector:	System
Type:	NO	Acquisition time:	2019-5-31 17:31:58 CST
Bottle number:	1	Method group:	peptide
Injection times:	1	Processing date:	2019-5-31 20:58:09 CST
Injection volume:	20.00 ul	Processing method:	0
Operation time:	30.0 Minutes	Channel name:	220.0 nm
		Channel annotation:	PDA 220.0 nm

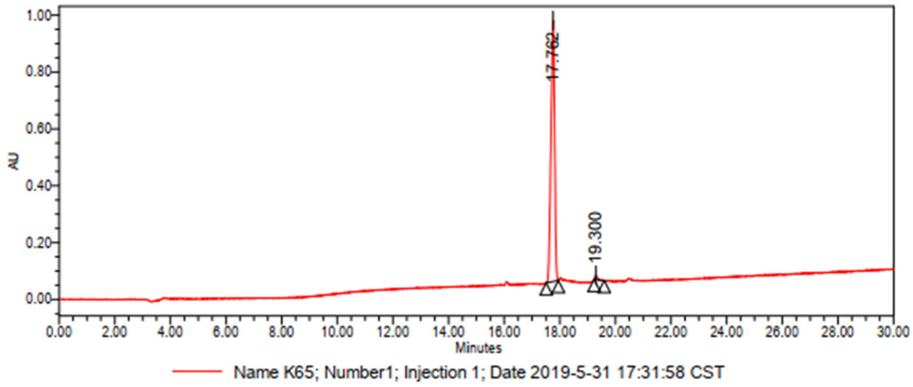

Channel: PDA 220.0 nm

	channel	retention time(min)	area	% area	peak height
1	PDA 220.0 nm	17.762	8323065	98.90	918299
2	PDA 220.0 nm	19.300	92528	1.10	15403

2.4. MS and HPLC data for Feleucin-K66

MS: expected mass: 952.5; Measured average mass: 951.7

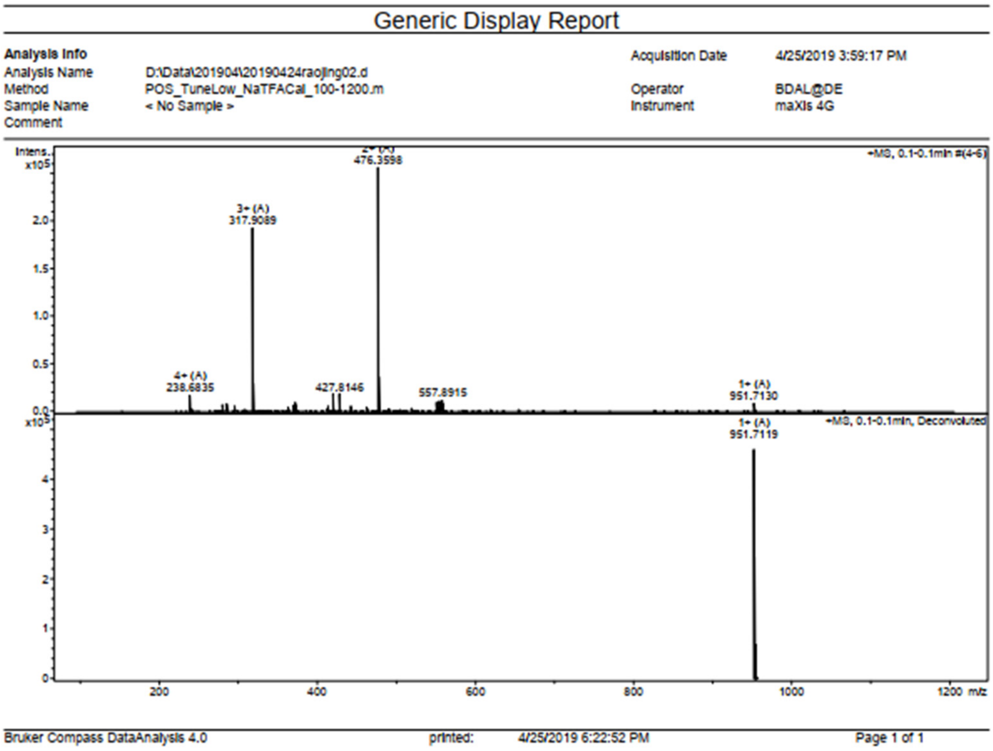

Customer Peptide HPLC Report Purity:96.25%

Sample Information

Name:	K66	Collector:	System
Type:	NO	Acquisition time:	2019-5-31 18:19:48 CST
Bottle number:	1	Method group:	peptide
Injection times:	1	Processing date:	2019-5-31 21:01:42 CST
Injection volume:	20.00 ul	Processing method:	0
Operation time:	30.0 Minutes	Channel name:	220.0 nm
		Channel annotation:	PDA 220.0 nm

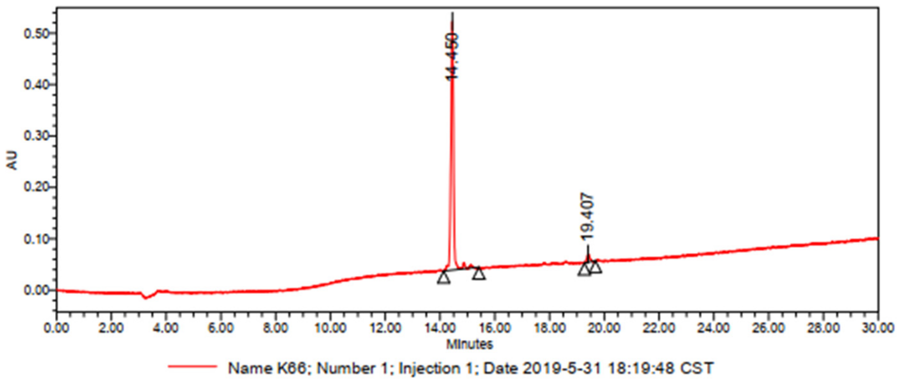

Channel: PDA 220.0 nm

	channel	retention time(min)	area	% area	peak height
1	PDA 220.0 nm	14.450	3213739	96.25	483436
2	PDA 220.0 nm	19.407	125260	3.75	15457

2.5. MS and HPLC data for Feleucin-K67

MS: expected mass: 1141.5; Measured average mass: 1140.8

Generic Display Report

**Analysis Info**		Acquisition Date	4/25/2019 4:00:29 PM
Analysis Name	D:\Data\201904\20190424raojing03.d		
Method	POS_TuneLow_NaTFACal_100-1200.m	Operator	BDAL@DE
Sample Name	< No Sample >	Instrument	maXis 4G
Comment			

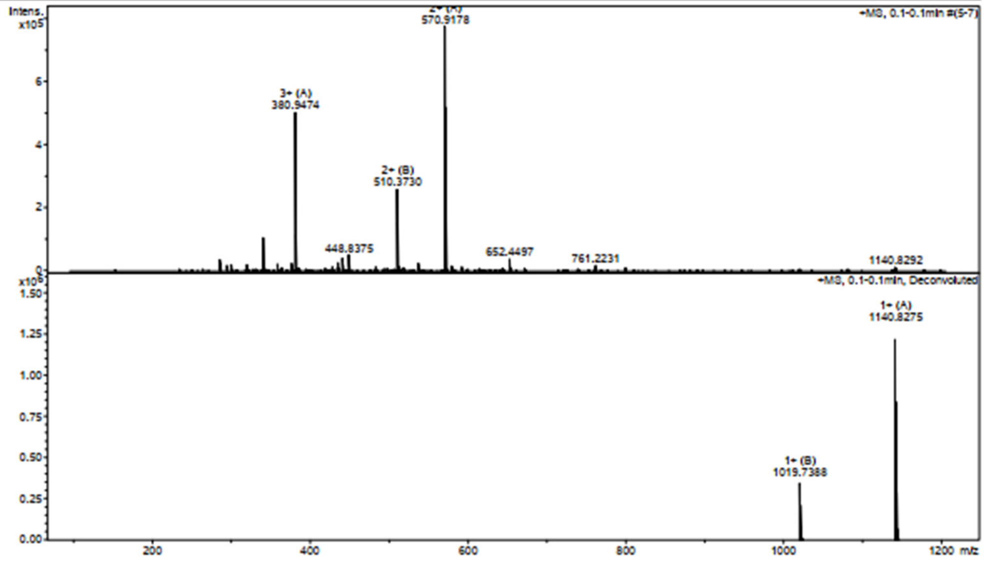

Bruker Compass DataAnalysis 4.0 printed: 4/25/2019 6:23:13 PM Page 1 of 1

Customer Peptide HPLC Report Purity:98.45%

Sample Information

Name:	K67	Collector:	System
Type:	NO	Acquisition time:	2019-5-31 20:02:52 CST
Bottle number:	1	Method group:	peptide
Injection times:	1	Processing date:	2019-5-31 21:03:01 CST
Injection volume:	20.00 ul	Processing method:	0
Operation time:	30.0 Minutes	Channel name:	220.0 nm
		Channel annotation:	PDA 220.0 nm

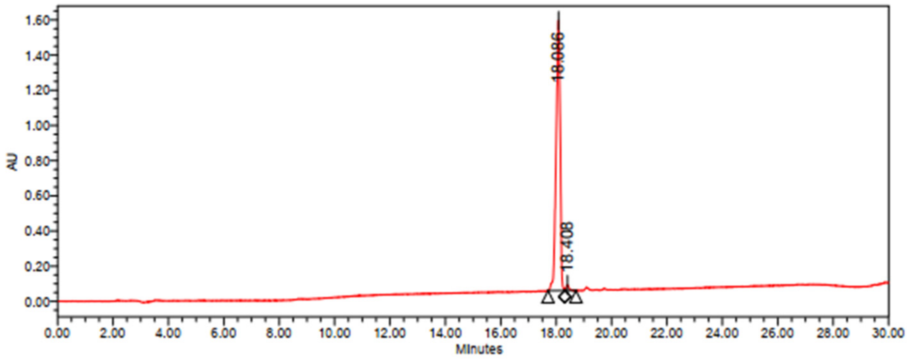

Name K67; Number 1; Injection 1; Date 2019-5-31 20:02:52 CST

Channel: PDA 220.0 nm

	channel	retention time(min)	area	% area	peak height
1	PDA 220.0 nm	18.086	15645465	98.45	1539079
2	PDA 220.0 nm	18.408	246630	1.55	33378

2.6. MS and HPLC data for Feleucin-K68

MS: expected mass: 1141.5; Measured average mass: 1140.8

Generic Display Report

**Analysis Info**		Acquisition Date	5/14/2019 4:57:47 PM
Analysis Name	D:\Data\201905\20190514raojing01.d		
Method	POS_TuneLow_NaTFACal_100-1200.m	Operator	BDAL@DE
Sample Name	< No Sample >	Instrument	maXis 4G
Comment			

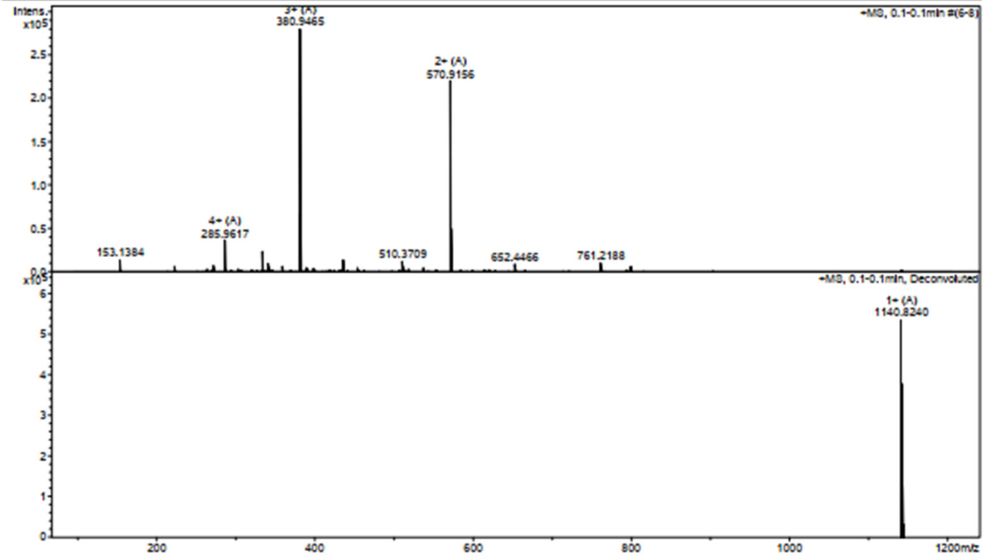

Bruker Compass DataAnalysis 4.0 printed: 5/15/2019 5:52:26 PM Page 1 of 1

Customer Peptide HPLC Report Purity:100.00%

Sample Information

Name:	K68	Collector:	System
Type:	NO	Acquisition time:	2019-5-31 20:47:18 CST
Bottle number:	1	Method group:	peptide
Injection times:	1	Processing date:	2019-5-31 21:18:16 CST
Injection volume:	20.00 ul	Processing method:	0
Operation time:	30.0 Minutes	Channel name:	220.0 nm
		Channel annotation:	PDA 220.0 nm

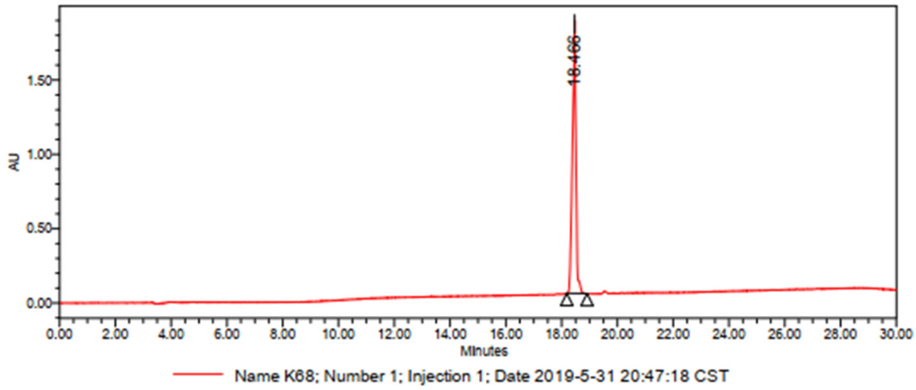

Channel: PDA 220.0 nm

	channel	retention time(min)	area	% area	peak height
1	PDA 220.0 nm	18.466	15471650	100.00	1833445

2.7. MS and HPLC data for Feleucin-K69

MS: expected mass: 1141.5; Measured average mass: 1140.8

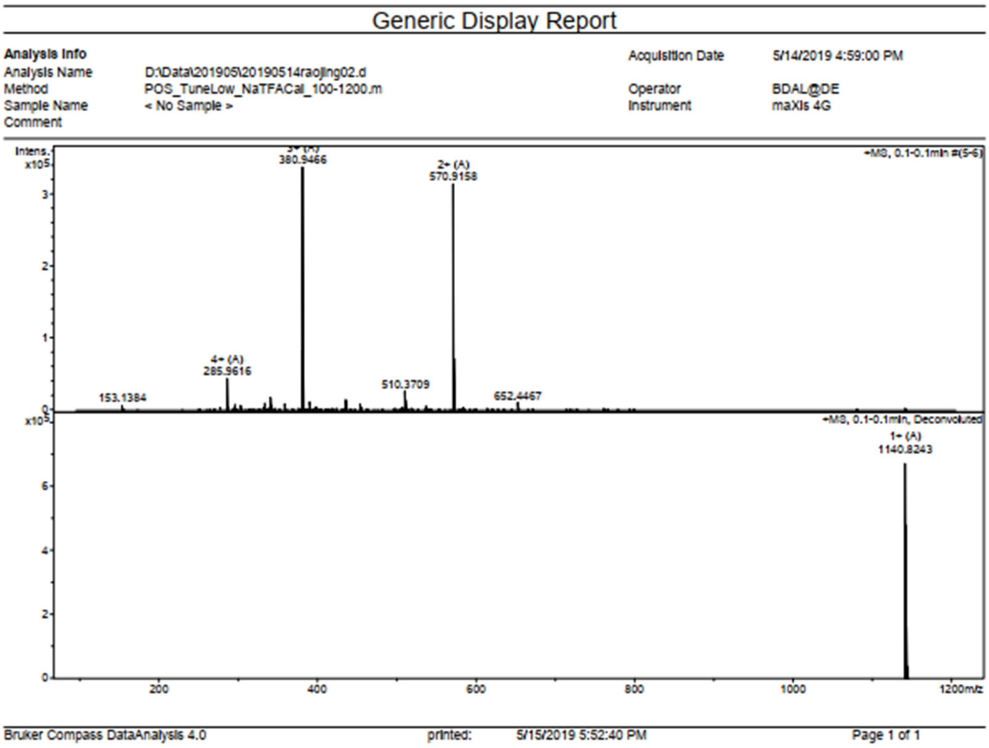

Customer Peptide HPLC Report   Purity:100.00%

Sample Information

Name:	K69	Collector:	System
Type:	未知	Acquisition time:	2019-6-13 17:33:55 CST
Bottle number:	1	Method group:	peptide
Injection times:	1	Processing date:	2019-6-15 10:03:59 CST
Injection volume:	20.00 ul	Processing method:	0
Operation time:	30.0 Minutes	Channel name:	220.0 nm
		Channel annotation:	PDA 220.0 nm

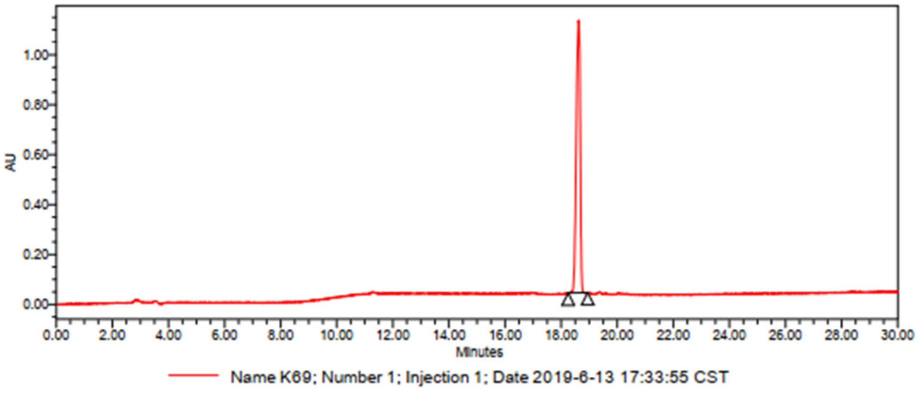

Channel: PDA 220.0 nm

	channel	retention time(min)	area	% area	peak height
1	PDA 220.0 nm	18.635	10688139	100.00	1089819

2.8. MS and HPLC data for Feleucin-K70

MS: expected mass: 1141.5; Measured average mass: 1140.8

Generic Display Report

Analysis Info			
Analysis Name	D:\Data\201905\20190514raojing03.d	Acquisition Date	5/14/2019 5:00:08 PM
Method	POS_TuneLow_NaTFACal_100-1200.m	Operator	BDAL@DE
Sample Name	< No Sample >	Instrument	maXis 4G
Comment			

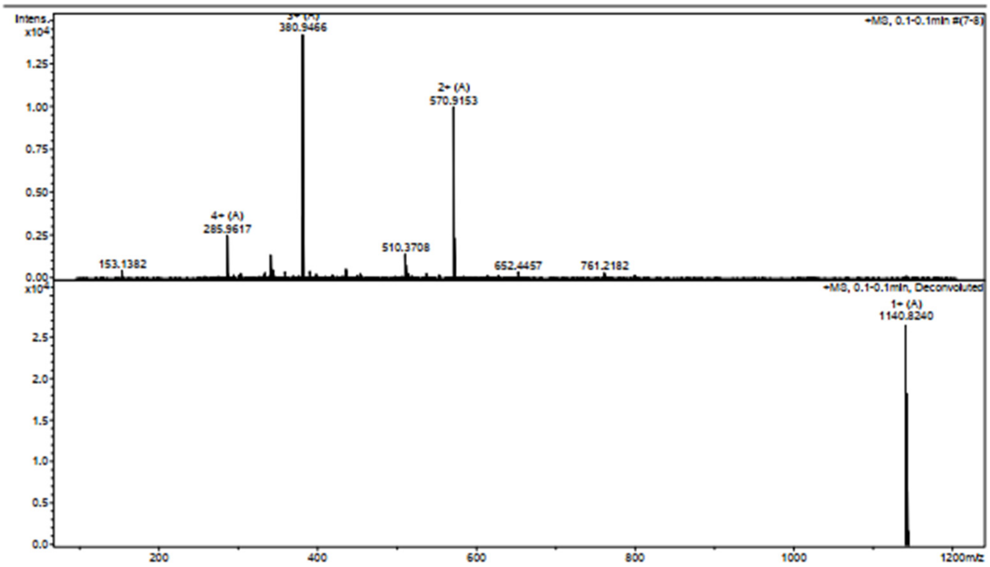

Bruker Compass DataAnalysis 4.0 printed: 5/15/2019 5:53:02 PM Page 1 of 1

Customer Peptide HPLC Report Purity:100.00%

Sample Information

Name:	K70	Collector:	System
Type:	NO	Acquisition time:	2019-6-13 19:28:33 CST
Bottle number:	1	Method group:	peptide
Injection times:	1	Processing date:	2019-6-15 10:06:13 CST
Injection volume:	20.00 ul	Processing method:	0
Operation time:	30.0 Minutes	Channel name:	220.0 nm
		Channel annotation:	PDA 220.0 nm

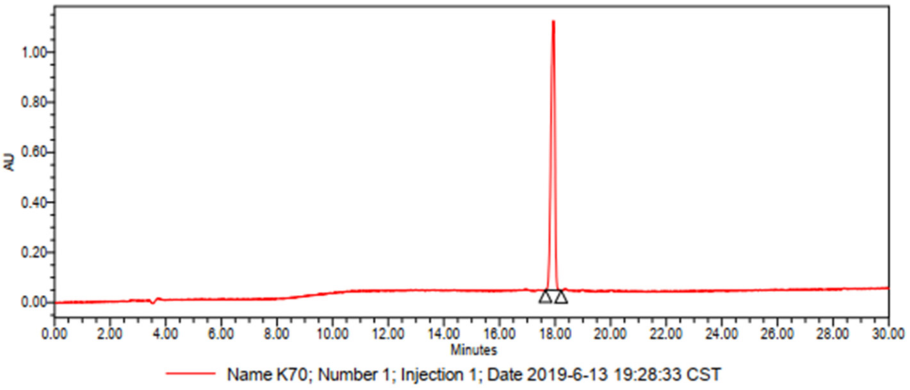

Channel: PDA 220.0 nm

	channel	retention time(min)	area	% area	peak height
1	PDA 220.0 nm	17.942	10441606	100.00	1077355

2.9. MS and HPLC data for Feleucin-K71

MS: expected mass: 1126.8; Measured average mass: 1125.8

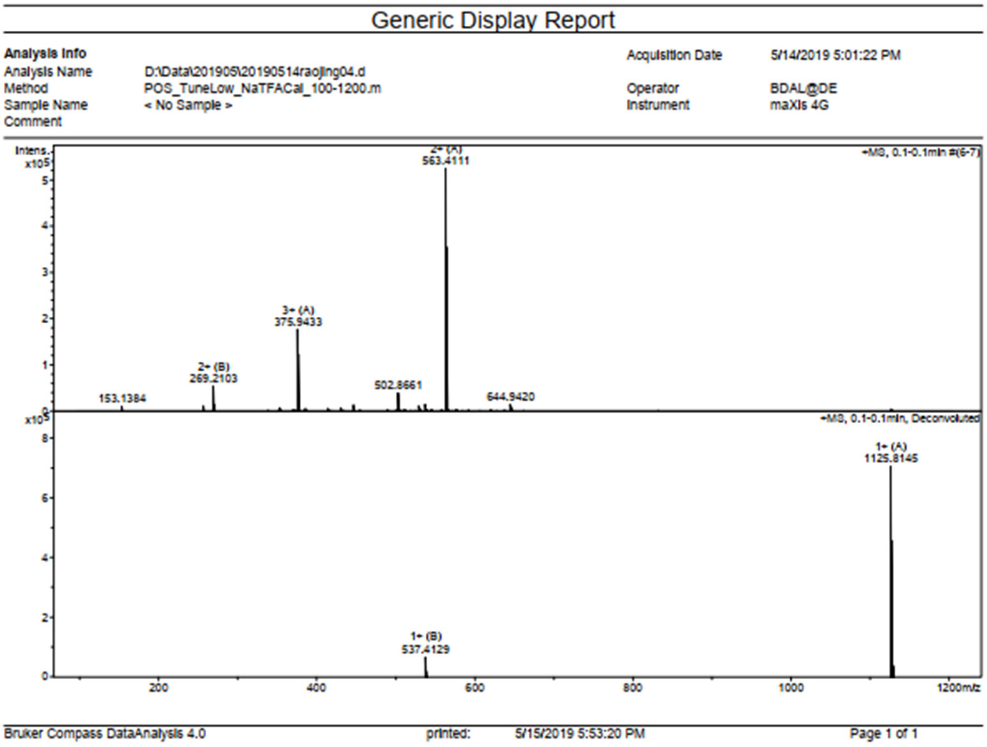

Customer Peptide HPLC Report Purity:100.00%

Sample Information

Name:	K71	Collector:	System
Type:	NO	Acquisition time:	2019-6-27 13:37:12 CST
Bottle number:	1	Method group:	peptide
Injection times:	1	Processing date:	2019-6-28 15:08:48 CST
Injection volume:	20.00 ul	Processing method:	0
Operation time:	30.0 Minutes	Channel name:	220.0 nm
		Channel annotation:	PDA 220.0 nm

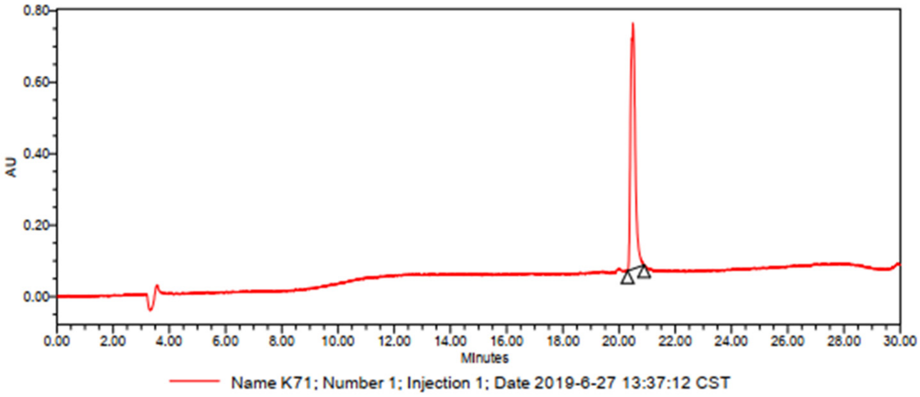

Channel: PDA 220.0 nm

	channel	retention time(min)	area	% area	peak height
1	PDA 220.0 nm	20.493	7843637	100.00	687935
